# Use of game fauna by Fulni-ô people in Northeastern Brazil: implications for conservation

**DOI:** 10.1186/s13002-020-00367-3

**Published:** 2020-04-17

**Authors:** Josivan Soares da Silva, André Luiz Borba do Nascimento, Rômulo Romeu Nóbrega Alves, Ulysses Paulino Albuquerque

**Affiliations:** 1 Programa de Pós-graduação em Etnobiologia e Conservação da Natureza, Departamento de Biologia, Universidade Federal Rural de Pernambuco, Recife, Brazil; 2 Departamento de Botânica, Laboratório de Ecologia e Evolução de Sistemas Socioecológicos, Universidade Federal de Pernambuco, Recife, Brazil; 3 Departamento de Zoologia, Universidade Estadual da Paraíba, Campina Grande, Brazil

**Keywords:** Bushmeat, Ethnozoology, Vertebrates, Traditional ecological knowledge

## Abstract

**Background:**

Due to the influence of several factors on the hunting of game meat, we investigated how the seasonality of the environment, the abundance, and the biomass of wild animals, as well as the proximity to these resources, can affect the hunting.

**Methods:**

The research was developed with the Fulni-ô people in the municipality of Águas Belas, Agreste of Pernambuco, Northeast of Brazil. In order to do this, we applied snowball sampling to select the participants. Data from potentially useful game species were obtained from lists and semi-structured interviews to register their particular kind of uses, capture periods (daytime, night, or both), preferences, and perceived abundance. The hunters who allowed their game meat captured to be weighed and identified were followed for 1 year.

**Results:**

Our records pointed to a vast repertoire of potentially hunting animals. However, we did not verify relationships between the abundance, seasonality, and biomass of the animals that were hunted by the Fulni-ô. We observed a total of 209,866 (kg) of game meat hunted in the studied group, belonging to 23 species, distributed in three taxonomic groups, the birds being the most representative group with 59% of total reported.

**Conclusion:**

Such consumption by the group is well below in terms of biomass when compared to other ethnic or local groups in other regions of Brazil, or in Caatinga areas, characterizing an activity much more of cultural character than subsistence. Also, the use of game meat among the Fulni-ô seems to be actively directed to the preferred species, suggesting that in the case of an urbanized indigenous community, where other sources of income are available, the demand for game meat is lower when compared to other ethnic groups.

## Background

The intensive use of animals for the supply of nutritional demands by local and traditional communities worldwide has attracted the attention of several researchers due to their effects on faunal stocks caused by overexploitation [1]. The consumption of game meat is considered the primary reason for game hunting, often occurring in developing countries with great wildlife richness, mainly in the African, Asian, and South American continents [1]. In Brazil, despite its legal restriction, hunting persists in different socio-cultural contexts [2–4], being allowed in subsistence conditions [5]. However, hunting of wild animals persists to a greater or lesser extent in all Brazilian biomes, being strongly associated with cultural aspects and the nutritional needs of the human groups involved [3].

Concerning indigenous peoples, this relationship becomes even stronger, contributing to the subsistence of these peoples, as well as to the maintenance of their cultural identity [6–8]. It is secured to them by the Brazilian constitution their rights to maintain their lands, way of life, and traditions [9]. For these peoples, scientific research in the Amazon has pointed out a large number of species with hunting importance that varies with their size, taxonomic group, and utilitarian purposes [2, 4, 10]. However, considering the semi-arid region, there is still little research on the dynamics of wildlife consumption by traditional communities in the Northeast region of Brazil [11, 12].

The hunting of wild animals involves a series of factors that may influence the choice of individual species to the detriment of others [2, 3, 10, 13–15]. The biological characteristics of species, such as abundance and biomass, for example, may direct the preference for specific prey among the hunters providing a higher post-activity yield [10]. Peres [2] observed that, in several places in the Amazon region, the rural population consumes between 9.6 and 23.5, birds and mammals, representing total estimated biomass of 67.173 to 164.692 tons and a yield of 36.392 to 89.224 tons of wild meat appropriate for consumption. Large animals tend to be more hunted and consequently more impacted than medium and small animals [2, 10]. Likewise, it is justified that taxa such as mammals and birds may be more in demand due to their biomass and abundance, respectively [1, 16].

In addition to the factors cited, climatic seasonality can affect the frequency of visitation to hunting areas [7] and the availability of these resources in the environment. These authors observed that the rainy season is considered the most favorable to hunt, due to the greater availability of food resources for the animals, easy identification of the traces left, and reduced risks of hunters being perceived. Conversely, in environments with strong climatic variations, such as the Caatinga, this activity may be more frequent in periods of seasonal drought, when the use of other resources becomes scarce [17].

Hunting in the Caatinga plays a strong socioeconomic role. Thus, the use of several vertebrate groups is an important source of protein for rural and urban communities [17]. For indigenous people living in these regions, hunting sometimes becomes a more frequent activity in relation to other activities involving animals, such as fishing, due to the drought regime [18]. In these areas, even cattle breeding and pasture areas, as well as plant crops, are strongly affected by droughts, which may make hunting a subsistence alternative [10, 12]. Another factor mentioned in the literature for the selection of game species is the proximity of human settlements to hunting areas. Studies indicate that this variable influences not only the species choices but also the amount of biomass acquired [11, 15]. Santos et al. [19], evaluating the effect of migration and incorporation on the medicine repertoire of the Truká indigenous people in the semi-arid region, that the proximity of other available resources, may favor the incorporation of new useful species into these communities. This distancing from the urban centers is related to a decrease in the availability of domesticated meat and may increase the demand for game meat.

Similarly, the authors argue that the proximity of useful resources decreases the costs of the search and investments of obtaining strategies [20]. However, Peres and Nascimento [10] point out that these meetings in the vicinity of the settlements may favor the capture of small animals that present a higher reproductive rate in relation to large animals. For the Fulni-ô people, seasonality is an important factor that marks most of the activities, which are performed by the opportunities that change between the seasons [21].

Thus, this research focused on the hunting of game meat among the Fulni-ô people living in the semi-arid region, being the first study carried out in an ethnic group strongly influenced by urbanization in the Brazilian Northeast. Thus, our objective was to verify the possible influences of environmental variables and biological characteristics of the species (as perceived by the people) on hunting behavior.

For this, we tested if species that are perceived as more abundant by these hunters are also more hunted. We expect that the amount of annual biomass caught (kg) of species considered more abundant will be significantly higher than that of species considered less abundant. Also, we verify if the proximity to the faunal resource makes it possible to obtain it. Therefore, we tested if the amount of biomass caught in the months of religious ceremony, in which the members of the group move to a settlement near the Ouricuri forest, is significantly higher than in other months. We also tested whether seasonality influences the hunting of game meat in the region, and we expect that the amount of biomass (kg) is significantly higher in the dry season than in the rainy season. Finally, we tested if there is a relation between the preference of game species by the hunters and the hunting of game meat. We expect that the most preferred game meat biomass will be higher than the least preferred ones.

## Methods

### Study area

The study was carried out in the Fulni-ô indigenous community, located in the municipality of Águas Belas (9° 06′ 45′′ S and 37° 07′ 15′′ W) in the state of Pernambuco. The Fulni-ô territory occupies 11,500 ha of the municipality of Águas Belas [22], presenting a Caatinga biome, with a hot and humid semi-arid climate with an annual average temperature of 24.5 °C. The vertebrate fauna in the Caatinga is composed of about 175 reptiles and amphibians, 510 birds, and 158 mammals.

The village presents tree divisions: the main village, the Xixiaclá village, and the sacred village (Ouricuri—used in a specific period of the year). According to information obtained from the health clinic of the village, about 3430 indigenous people live in the main village, and about 100 live in the Xixiaxlá village located near the Ouricuri forest. Their dialect characterizes the members of the Fulni-ô group, “Yatê,” belonging to the Macro-Jê language group, besides the Portuguese language, and by their annual religious ceremony called “Ouricuri.” During the ceremony, the people migrate from September to December to a region distant from the main village, surrounded by Caatinga vegetation, which forms one of the fewest native forest fragments in the region [12, 23, 24]. This religious period is a time of ethnic seclusion in another village (Ouricuri village) in which the Fulni-ô suspend all external activities and dedicate themselves exclusively to religious demands.

The main village is located a few meters from the town of Águas Belas and also has urban buildings. Among economic activities, Campos [18] emphasizes subsistence and commercial agriculture, handicrafts, rental of land, artistic presentations, and works in institutions in the municipality and other cities. Also, in their rites or cultural presentations, they use body paintings extracted from local plants and, unlike other ethnic groups, they use hats made from the straw of the Ouricuri palm (*Syagrus coronata* (Becc.) Becc) instead of headdresses. Regarding the faunistic use among them, Campos [18] pointed out that fishing in the year 2012 is the predominant activity performed in the village. However, given the climatic seasonality in the region, observations during the study period indicate that fishing activity is almost incipient, since areas that allow such activity are practically nonexistent, information that is supported locally. Thus, with regard to fauna, hunting among the Fulni-ô people is the most frequent activity today. It usually occurs in the vicinity of the village and involves only men [18]. However, due to the proximity to the urban center of the municipality, hunting of wild animals can be replaced by the acquisition of non-wild animal protein acquired in conventional fairs and markets.

The “warriors,” as the hunters are called, see in this activity an important source of obtaining resources for food and religious purposes, in addition to their by-products being used in local medicine and often used for making handicrafts. The latter contains mainly by-products of birds such as feathers that are used to make the headdresses, arrows, and decorative articles that often refer to the white man’s vision of what is “indigenous” but which are not part of Aldeia’s traditional repertoire [25]. Also, other materials of animal origin, such as teeth, skins, and claws, have been observed in situ and are frequently used in the making of these craft artifacts. Exchanges can also acquire them in cultural encounters with other ethnicities. The production of handicrafts is one of the essential activities of income generation for the Fulni-ô, and many of the materials used are of plant origin, mainly the Ouricuri palm (*S. coronata*) with which they make mats, baskets, hats, among others.

The environmental conditions of the region favor the choice of areas for hunting and gathering of vegetal resources. Nevertheless, many of these areas have suffered successive disturbances such as burning, deforestation, and inadequate use of the soil, which leads them to develop mechanisms that guarantee their physical and cultural existence [21]. The Fulni-ô hunting may occur in groups or individually, usually depending on the chosen taxon. We observed in situ that the foraging strategies among the Fulni-ô people are facilitated by utilizing motorcycles or bicycles to the selected areas. Collective hunts are usually planned hours or days earlier among the hunters involved. For animals like birds, collective hunting ends up being more frequent, usually occurring at night time with the aid of lanterns to immobilize the animals and firearms, or associating the use of the firearms and the assistance of dogs in daytime hunting as well. It is also common to use techniques like fall traps that are generally employed for medium and small mammals.

### Data collection

The first contact of our research group occurred in the year 2014 for the recognition of the study area and the presentation of proposals. This contact and the selection of the study area occurred due to a relationship already established by members of our team who researched the Village in previous years [23, 26, 27]. This relationship facilitated the access and the establishment of a relationship of confidence to obtain the information from the hunters. Subsequently, the project adjusted to the Fulni-ô was presented to the leaders of the Village, in the person of the shaman, representative of the cacique at the time, when the proposal and stages of the research were exposed. After the explanation of the objectives of the study, we were granted the prior consent term with the approval of the leadership and authorization of the local FUNAI. The proposal was also approved by the Human Research Ethics Committee of the University of Pernambuco (CAAE: 94508218.0.0000.5207), and the project is registered in the National System for Genetic Heritage Management and Associated Traditional Knowledge - SISGEN (No. AA869DC) in compliance with the provisions of Law 13,123 of 2015 and its regulations.

The group of the focus of the present research was people locally recognized as hunters with a minimum age of 18 years. The identification of the participants was achieved through the application of the “snowball” technique (see description in [28]). Data from potentially useful game species were obtained from free lists and semi-structured interviews [28], and their particular kind of uses, capture periods (daytime, night, or both), preferences, and perceived abundance were recorded. We recorded at the time of the interviews which of the animals cited by each hunter were preferred. The perceived abundance was recorded individually using the “punctuation exercise” technique [29], in which respondents attributed values between zero and ten, “zero” being equivalent to the disappearance of the species in the region and “ten” for the cases of very abundant animals.

The monitoring of the hunting of game species was carried out monthly between June 2015 and July 2016. For this purpose, five hunters were selected from the indication of a key informant, due to their experience or frequency in hunting activities, being trained to register the hunted animals in the absence of researchers. These informants resided in distinct areas of the Village in order to facilitate the registration of hunting by as many hunters as possible. Thus, the common name of the species, the number of animals, the period of the hunting, and their respective biomass (kg) were recorded. Of the 43 hunters approached in the first stage of the survey of useful species in the region, 20 aged 18 to 56 years old, allowed the registration and measurement of their games during the period of the research. The captured animals were weighed using a portable scale (up to 50 kg). To record information about local seasonality, we use the region’s rainfall record as a proxy on a 10-year time scale. For this, we consulted the online database of the website of the Pernambuco and Water and Climate Agency (APAC).

The identification of the animals was performed with the assistance of a specialist from the Federal University of Paraíba through photographs taken by the hunters or the researchers at the time of the capture of the animals. Additionally, for animals that were not possible to identify by photographic record, a checklist [29] (see supplementary file 1) containing images of the possible species cited by the interviewees was created. Images of animals not occurring in the region were also added to avoid possible influences on hunters’ indications. This technique is commonly used mainly in cases where there are difficulties in obtaining parts of the animals after their capture and use by the hunters. Scientific records on the fauna occurring in the Caatinga environments were used for the elaboration of this checklist [12, 17, 30, 31], which were later presented to the people to confirm their occurrence in the locality. The classification of species in relation to their conservation status was obtained by consulting the International Union for Conservation of Nature (IUCN) (iucnredlist.org) and the Red Book of Brazilian Endangered Fauna (Brazilian Red List).

### Data analysis

In order to analyze if the hunting of game meat was influenced by seasonality, a simple linear regression was performed between the number of animals hunted monthly and the monthly cumulative precipitation data [32], as well as between the total weight of the hunted animals (kg) monthly and the cumulative monthly precipitation. Also, to assess whether there were dissimilarities among the number of individuals hunted per species seasonally, a similarity matrix was built using the Bray-Curtis index. Then the PERMANOVA analysis was used to check for dissimilarities between the rainy months and dry months (considering the APAC classification) [32]. Finally, in order to test whether there were species that could be indicative of the dry period or the rainy season according to the number of individuals hunted, the analysis of indicator species was performed.

In order to analyze if there were differences in the hunting of game meat between the period of Ouricuri and the rest of the year, a *t* test was performed between the number of individuals hunted during the ritual and non-ritual months, as well as between the weight of individuals hunted during the ritual and those hunted in other periods. In addition, to evaluate whether there were dissimilarities between the number of individuals hunted per species between the Ouricuri period and the rest of the year, a similarity matrix was built using the Bray-Curtis index. Then the PERMANOVA analysis was applied to verify if there were dissimilarities between the months of the ritual and the other months of the year. Finally, in order to determine if there were species that could be indicative of the period of Ouricuri according to the number of individuals hunted, the analysis of indicator species (ISA) was performed.

In order to evaluate if species-perceived abundance influenced hunting, a generalized linear model (GLM) was applied using the “quasipoisson” family between the number of individuals hunted during the year by species and the perceived abundance average. The “quasipoisson” family was used on the species’ perceived abundance data in order to ensure greater control of the degrees of freedom. The same analysis (GLM) was performed considering the total weight of the game obtained during the year by species and the average abundance perceived. Finally, to check if the species cited as preferred were more hunted than the other species, the Mann-Whitney test was performed for independent samples between the annual number of hunted individuals and the total annual weight of preferred and non-preferred hunted individuals.

All analyses were performed using the software R version 3.4.1 [33]., in which all results whose *p* < 0.05 were considered significant were considered. The normality of the data was verified through the Shapiro-Wilk test. For the analysis of PERMANOVA, the Vegan package was used [34], and the “indicspecies” package was used for the analysis of indicator species [35].

## Results

Among the 64 potentially useful animals indicated from the semi-structured interviews (supplementary file 2), 22 species were frequently hunted within the period of the present study. A total of 673 animals were hunted between the years 2015 and 2016 by the Fulni-ô, representing 209,866 (kg) of game meat hunted. Among the animals, 77% were hunted during the day, and birds were the taxonomic group that presented 59% of the game, with the highest rate for *Zenaida auriculata* (Des Murs., 1847) (26,935 kg). Mammals followed birds with 27%, among which the most hunted was *Galea spixii* (Wagler, 1831) (19,775 kg). Reptiles came subsequently with 14%, and the species *Salvator merianae* (Duméril & Bibron, 1839) was the most hunted within this group (79,100 kg) (Table 1).

**Table 1:** Biomass and perceived abundance of game fauna preferred among the Fulni-ô people in NE Brazil and conservation status. *Legend: IUCN Red List categories: LC - Least Concern, NT - Near threatened, NI - Not Included.**Legend: Brazilian Red List: LC - Least Concern, VU -Vulnerable, EN - In Danger

Common name	Group/species	Number of individuals hunted in the year	Total biomass (g) of game meat in the year	Biomass captured in religion ceremony (g)	Perceived abundance	Cited as preferred?	Capture period	IUCN*	Brazilian Red List**
**Mammals**									
Mocó	*Kerodon rupestris* (Wied-Neuwied, 1820)	13	3953	–	6.68	No	Daytime	LC	VU
Cambambá	*Conepatus semistriatus* (Boddaert, 1785)	1	2000	2000	5.67	No	Night	NT	LC
Gato-pintado	*Leopardus tigrinus* (Schreber, 1775)	1	4500	4500	3	No	Daytime and night	NT	EN
Peba	*Euphractus sexcinctus***(**Linnaeus, 1758)	1	4000	–	3.45	No	Night	LC	LC
Cabudo	*Thrichomys apereoides* (Lund, 1839)	1	200	**–**	**7**	No	Daytime	LC	LC
Preá	*Galea spixii* (Wagler, 1831)	150	19,775	6400	3.68	Yes	Daytime	NT	LC
**Birds**									
Rabaçam	*Zenaida auriculata* (Des Murs, 1847)	226	26,935	19,490	6.69	Yes	Daytime	LC	LC
Rolinha-pivó	*Columbina minuta* (Linnaeus, 1766)	69	2870	100	8.17	Yes	Daytime	LC	LC
Codorna	*Nothura maculosa* (Temminck,1815)	62	12,610	5730	6.18	Yes	Daytime	LC	LC
Carcará	*Caracara plancus* (J. F. Miller, 1777)	24	13,530	4100	6.88	No	Daytime	LC	LC
Asa Branca	*Patagioenas picazuro* (Temminck, 1813)	10	2483	–	2.6	No	Daytime	LC	LC
Gavião	*Elanus leucurus* (Vieillot, 1818)	5	5490	4500	5.62	No	Daytime	LC	LC
Lambú	*Crypturellus parvirostris***(**Wagler, 1827)	5	1140	1000	5.89	Yes	Daytime	LC	LC
Codorniz	*Nothura boraquira* (von Spix, 1825)	5	1485	200	5.36	Yes	Daytime	LC	LC
Patori	*Amazonetta brasiliensis* (Gmelin, 1789)	4	5030	–	2	No	Daytime and night	LC	LC
Sariema	*Cariama cristata* (Linnaeus, 1766)	2	3020	–	3.25	No	Daytime	LC	LC
Anum-branco	*Guira guira* (Gmelin, 1788)	2	290	–	9	No	Daytime	LC	LC
Jaçanã	*Jacana jacana* (Linnaeus, 1766)	1	250	250	6	No	Daytime	LC	LC
Socó	*Tigrisoma lineatum* (Boddaert, 1783)	1	1100	–	3	No	Daytime	LC	LC
**Reptiles**									
Teiú	*Salvator merianae* (Duméril & Bibron, 1839)	72	79,100	32,200	6.65	Yes	Daytime	LC	LC
Camaleão	*Iguana iguana* (Linnaeus, 1758)	15	10,105	1005	6.81	Yes	Daytime	LC	LC
Jibóia	*Boa constrictor* Linnaeus, 1758	3	10,000	10,000	5.6	No	Daytime and night	NI	LC

### Perception of species abundance and game animals hunted

The perceived abundance of species did not explain the number of individuals hunted per month (estimated = 0.18, standard error = 0.24, *t* = 0.766, *p* > 0.05) or hunting biomass (estimated = 0.17, standard error = 0.21, *t* = 0.805, *p* > 0.05). These results show that perceived abundance is not a factor that influences the hunting of game meat.

### Influences of the Ouricuri ritual on the hunting of game meat

Similarly, we did not observe a significant difference (*t* = 1.49, *p* > 0.05) between the number of individuals hunted in the Ouricuri rituals ($\overline{\mathrm{X}}$ = 85, dp = 51.02) and the other months of the year ($\overline{\mathrm{X}}$ = 38.62, dp = 50.19), and there was no difference (*t* = − 1.86, *p* > 0.05) between the biomass hunted during the Ouricuri ritual ($\overline{\mathrm{X}}$ = 22.87, dp = 6.19), and after the ritual ($\overline{\mathrm{X}}$ = 13.58, dp = 11.07). Furthermore, we did not find any variation between the periods mentioned above in relation to the number of hunted species (*R*^2^ = 0.13, *F* = 1.53, *p* > 0.05). Finally, the analysis of indicator species found that only *Z. auriculata* is significantly associated with the dry period in the region that corresponds to the same period of the Ouricuri ritual (Stat = 0.96, *p* < 0.05), being it predominantly hunted in this period. These results show us that the migratory event related to the Ouricuri ritual is not a factor that generates variation in the amount of game meat hunted.

### Seasonal variations in the hunting of game meat

The rainfall variation during the year of observation neither explained the number of individuals hunted per month (*R*^2^ = 0.04, *F* = 0.471, *p* > 0.05) nor the biomass (*R*^2^ = 0.01, *F* = 0.126; *p* > 0.05). In addition, there was no seasonal variation (dry and rainy) in the number of individuals hunted per species (*R*^2^ = 0.05, *F* = 0.62, *p* > 0.05). Finally, the analysis of indicator species found that only *Patagioenas picazuro* (Temminck, 1813) is significantly associated with the rainy season (Stat = 0.857, *p* < 0.05), and it is possibly the most hunted in this period.

### Preference and hunting of game meat

**Fig. 1:**
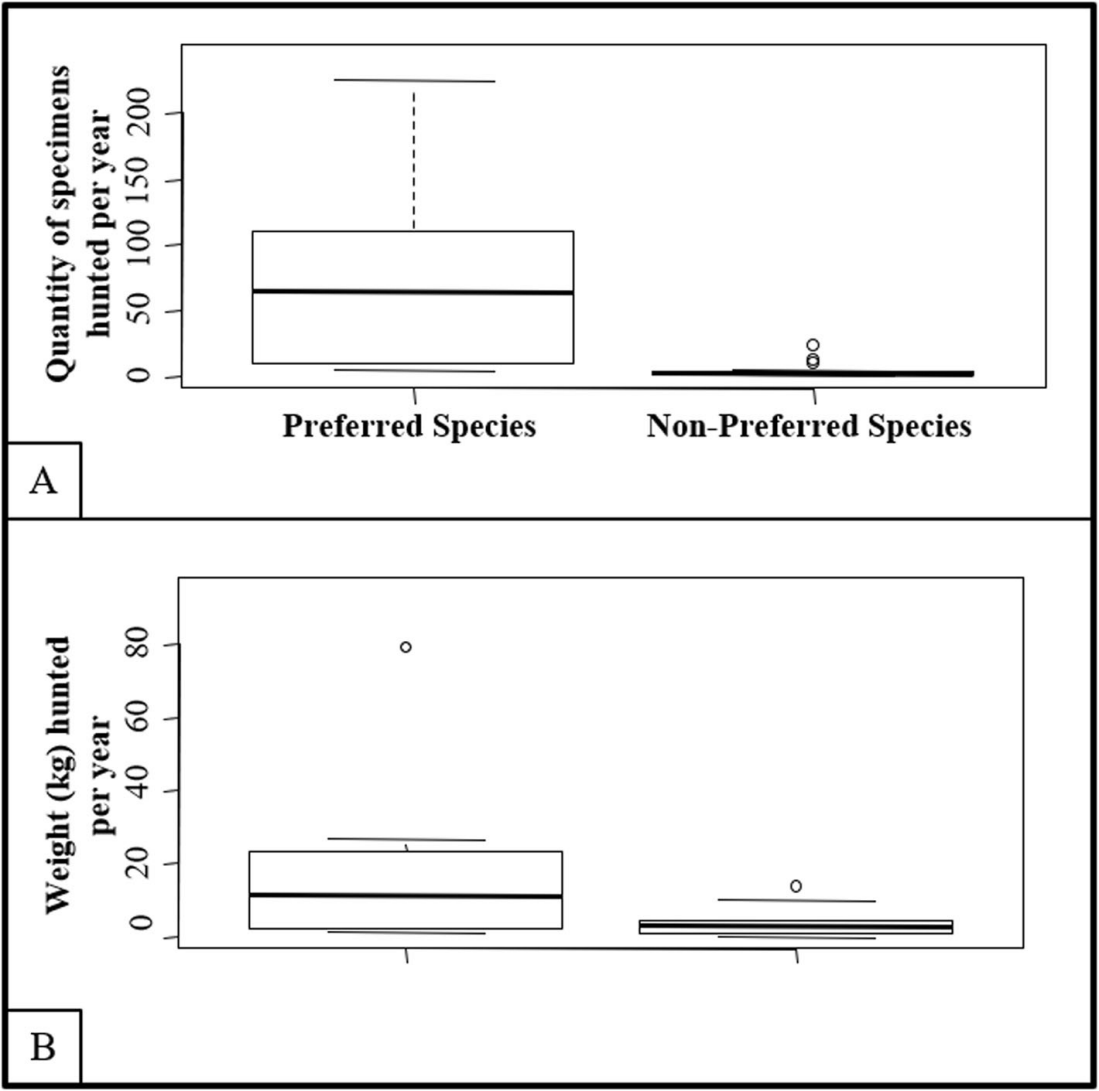
Annual comparison of the consumption of game meat of the species mentioned as preferred and the other species recorded in the indigenous village Fulni-ô, Northeast Brazil. **a** Number of individuals hunted by species. **b** Total weight of individuals hunted by species

We found significant differences (*W* = 112, *p* < 0.001) between the number of hunted individuals belonging to species cited as preferred by the informants and the other hunted species (Fig. 1). Also, there was a difference (*W* = 91, *p* < 0.05) between the biomass belonging to the species cited as preferred by the informants and the other hunted species (Fig. 1). These results allow us to infer that the species cited as preferred by the informants are also the most hunted in the community.

## Discussion

Although the low number of volunteers monitored hunters in the second stage of hunting monitoring (*n* = 20), we believe that these data represent the current scenario of hunting among the most active hunters that allowed the collection of such information. The results on the number of animals and taxa hunted diverge from other studies regarding the annual rate of extraction of these resources in places with high faunistic diversity, such as the Amazon region [2, 10, 36], as well as in other tropical regions such as many African countries [37–40]. These differences in relation to the Amazon, for example, may be directly related to the faunistic composition and its size and the nutritional demands of the region [4, 10]. Even when compared to studies of wild animal consumption in the Caatinga region [5, 41], the number of animals hunted is higher than that reported for the Fulni-ô, which may be indicative of a lower demand for game in relation to other communities in the northeast region [5, 12, 17, 31].

These differences in the hunting of game meat observed for the Funi-ô in relation to other ethnic groups lead us to infer that hunting activity is much more focused on local cultural maintenance than a subsistence strategy. That low wild meat demand may suggest that hunters target their strategies at species whose attributes for choice include cultural aspects, such as the taste of meat or taboos. Campos [18] supports our idea by suggesting that hunting for this group is a food supplement for a few residential units. The author also argues that hunting is practiced for recreational purposes, domestic breeding, and the use of non-edible animals, such as some species of birds. This recent increase in wildlife utilization by the Fulni-ô caused by strong drought regimes, which consequently affects fishing activity in the region, can also be interpreted as a temporal reflection that may or may not change over time, depending on the availability of other natural resources. This characteristic change in the ways of obtaining natural resources was pointed out by Francesconi et al. [42], who argued based on their findings that hunting intensity may reflect a specific context of these communities in space and time or a cultural fusion of subsistence activities. Similarly, McNamana et al. [43] reinforce the idea that preference for game meat may not be fixed, varying according to the circumstances experienced by such hunters.

The cultural attributes, such as beliefs, attitudes, and social norms, are present and were equally important together with economic motivations for the hunting of wild animals in primarily urban areas [14]. Besides that, other factors that may reinforce the low dependency of the Fulni-ô on game meat is the involvement of hunters in other external subsistence activities and the ease of obtaining non-wild animal protein, due to the proximity to the urban centers. Fita et al. [44] point out that the decrease in hunting activity may be a result of the increase of alternative productions of consumption and income generation. This possibility of external alternatives is similarly highlighted in the study by Francesconi et al. [42], mainly among younger hunters who migrate more frequently to urban areas. This migration or the urbanization processes that local/traditional communities are subjected to, despite contributing to the well-being and providing opportunities for education and access to health, is pointed out by the authors as a factor that favors habitat fragmentation in their territories, which diminishes traditional sources of subsistence [45]. The latter author also points out that such urbanization processes can also contribute to the exclusion, invisibility, and violence of these peoples in addition to influencing the detachment from their traditional practices. These factors also imply less involvement of hunters in this activity [46–48], which leads the indigenous people to develop these practices in their free time. These factors are suggested in the literature as potential modelers of hunting practices due to the lower cost of obtaining the animal protein.

Likewise, most studies report a higher frequency of hunting on mammalian species, followed by birds and reptiles [10, 46], that we did not observe in our findings. The preference for mammals is not only due to their size or availability but also to the taste of the protein in relation to the other taxa [8, 10, 15, 41]. The most common mammal species observed in our research are mainly small animals, such as *G. spixii*. The high frequency of use of these animals can be considered an adjustment of the hunters to the reduction of the most sought species [20, 46]. Moreover, records in the literature point out that these small animals are less susceptible to hunting because of their high reproduction rate [2] and are commonly abundant in more anthropic areas [20].

On the other hand, it is important to point out that this trend towards *G. spixii* may also indicate a transfer of possible pressures of use in relation to other taxa of medium and large sizes [17, 41]. This requires the hunter to increase the effort to capture more of these animals to meet their demands. In addition, the defaunation that has been occurring in the semi-arid region may also be a factor for this transfer for the use of other species [17, 41]. This targeting of small species may indeed be a reflection of the decline of preferred species in the vicinity of human settlements, inducing hunters to search for animals other than their primary choices [17, 42].

The high rate of bird reported in our study is supported by other records in the literature that indicate the taxon as one of the most sought after by local communities in the Caatinga [5, 41, 47]. However, the high frequency of avifauna caught among the Fulni-ô should not only be a consequence of their high abundance but also of a greater efficiency in their attainment, since much of the incursions are carried out in groups and by applying a combination of strategies that optimize the success of the activity. This higher occurrence in the capture of birds, especially *Z. auriculata*, by members of this ethnicity, if interpreted in terms of the benefits generated after capture, reinforces the local importance of the possibility of multiple uses of these animals to the detriment of other less versatile species. This versatility of use may justify the fact that many of these birds are caught for non-food purposes, especially between March and April, the period of highest intensity in the artisanal confection in the Village. These are obtained exclusively for the extraction of their by-products with the purpose of making handcrafted artifacts [21], such as headdresses and arrows. Silva et al. [25], when analyzing the artisanal production of the Fulni-ô natives, also identified several artifacts that are made aiming at the external market demand, which are more related to the white man’s perception of indigenous peoples. However, the need of the market and the contact with other ethnic groups can cause the indigenous people to adjust their strategies of sale. Regarding the reptiles hunted in the studied region, the high rate of capture of *S. merianae* among the others reported may be mainly related to the return relative to biomass. In addition, like the birds, it may be related to the better use of its byproducts for local medicine, for example. This trend has been recorded in other researches that show that this lizard species is one of the most consumed in several regions of Latin America, and its lard is often used in the treatment of inflammation [49–52].

Regarding seasonality, the migratory event related to the ritual of Ouricuri (September to December), which increases the contact of Fulni-ô members with natural resources, did not show a significant variation in the composition of the fauna hunted in the region. This contradicts our expectations and other studies that observed that the proximity of foraging areas is an important factor for the increase in the use of natural resources [5, 53]. This intensification of faunal use was verified by Van Vliet and Nasi [53] in Northeast of Gabon, Africa, where they observed a temporal and spatial variation in the period of drought in the region during the same period of the local religious ceremony. Conversely, the ceremony of the Fulni-ô occurs, as already mentioned, in a longer period of drought, which is a certain way that limits the encounter with animals of other taxa and favors the encounter of species of birds, mainly migratory ones like *Z. auriculata.* This species was pointed out by Mendonça et al. [5] as one of the most hunted, which justifies the abovementioned species targeting due to their high abundance during periods of scarcity from other hunting sources. However, we cannot disregard the limitation on access to information during the reclusion period for the performance of the Ouricuri religious ceremony, in which this access is allowed only to members of the ethnic group. During this period, information about hunted animals was collected only by hunters who assisted us in the research.

Another relevant issue among the Fulni-ô is that most species were hunted during the daytime. This may also be a reflection of the very legality of the hunting activity for indigenous groups. Therefore, since hunting is an integral part of these cultural reaffirmation actions, it is expected that it will be performed more frequently in shifts that pose fewer risks to hunters. This safety of daytime hunting in relation to nocturnal hunting, due to better visibility of the foraging area and the greater presence of fauna preferred by the natives, may be a determining factor in the hunting practices in the Village. Night hunting is reported in the literature as a more costly practice, with less prey availability [53], which requires different strategies that are usually applied in combination and facilitate visibility reducing risks [15].

## Conclusion

Our results support previous studies carried out in the Caatinga region in which was observed a higher avifauna catch rate, although the low rate of vertebrate hunting observed here can be interpreted as a strong contrast between the demand of game meat from other communities and Fulni-ô ethnicity. Likewise, the variables abundance, biomass, and the proximity of the resource did not present strong influences in relation to the species hunted in the region. This allows us to infer that the use of game meat in the Village has a strong cultural aspect of maintenance and reaffirmation of the indigenous identity and not only of subsistence. This confrontation of the Fulni-ô cultural practice also can be a response to the strong influence of urbanization and the external income opportunities that allow the use of non-traditional faunal resources in the markets near the Village. This lower dependence of the resource as subsistence can be reinforced by the directing of the hunt to the species pointed out as the ones that are preferred by the hunters. Thus, the data presented here help to understand the dynamics of how this cultural practice occurs in the face of environmental processes such as scarcity of resources, urbanization, and aspects that are inherent to hunters, such as preferences and strategies adopted to guarantee a satisfactory return of the hunting practice.

On the other hand, it is also important to note that the cultural resistance of the Fulni-ô members is also supported by the preservation of their native language, their religious ceremony, and their different relationships with natural resources. Thus, ethnicity presents important contexts to be investigated by future research in relation to possible cultural adjustments due to socioenvironmental factors. Therefore, we emphasize the importance of integrating not only environmental or biological variables of the target species, but also cultural and social aspects about the hunting practices of traditional communities. Such understanding is also fundamental concerning to the food security of these peoples, especially in the current global scenario in which we live in a pandemic caused by the COVID-19 virus, whose origin and spread may have been influenced by the cultural habit of consuming wild species. From the perspective of conservation, the information presented here helps to evaluate the natural stocks of the local fauna, emphasizing the importance of integrating these communities into the management processes through strategies that stimulate such involvement, and supports the knowledge of the practice to be transmitted to the following generations.

## Supplementary information

**Additional file 1.** A checklist containing images of the possible species cited by the interviewees

**Additional file 2.** List of potentially useful game fauna indicated by Fulni-ô people in NE Brazil and conservation status.

**Additional file 3.**

**Additional file 4.**

**Additional file 5.**

## Data Availability

The datasets generated during and/or analyzed during the current study are available from the corresponding author on reasonable request.
