# Supplementary figures and images for: Use of game fauna by Fulni-ô people in Northeastern Brazil: implications for conservation
Source: J Ethnobiol Ethnomed. 2020 Apr 17;16:18. doi: 10.1186/s13002-020-00367-3 (PMC7164412; doi:10.1186/s13002-020-00367-3)

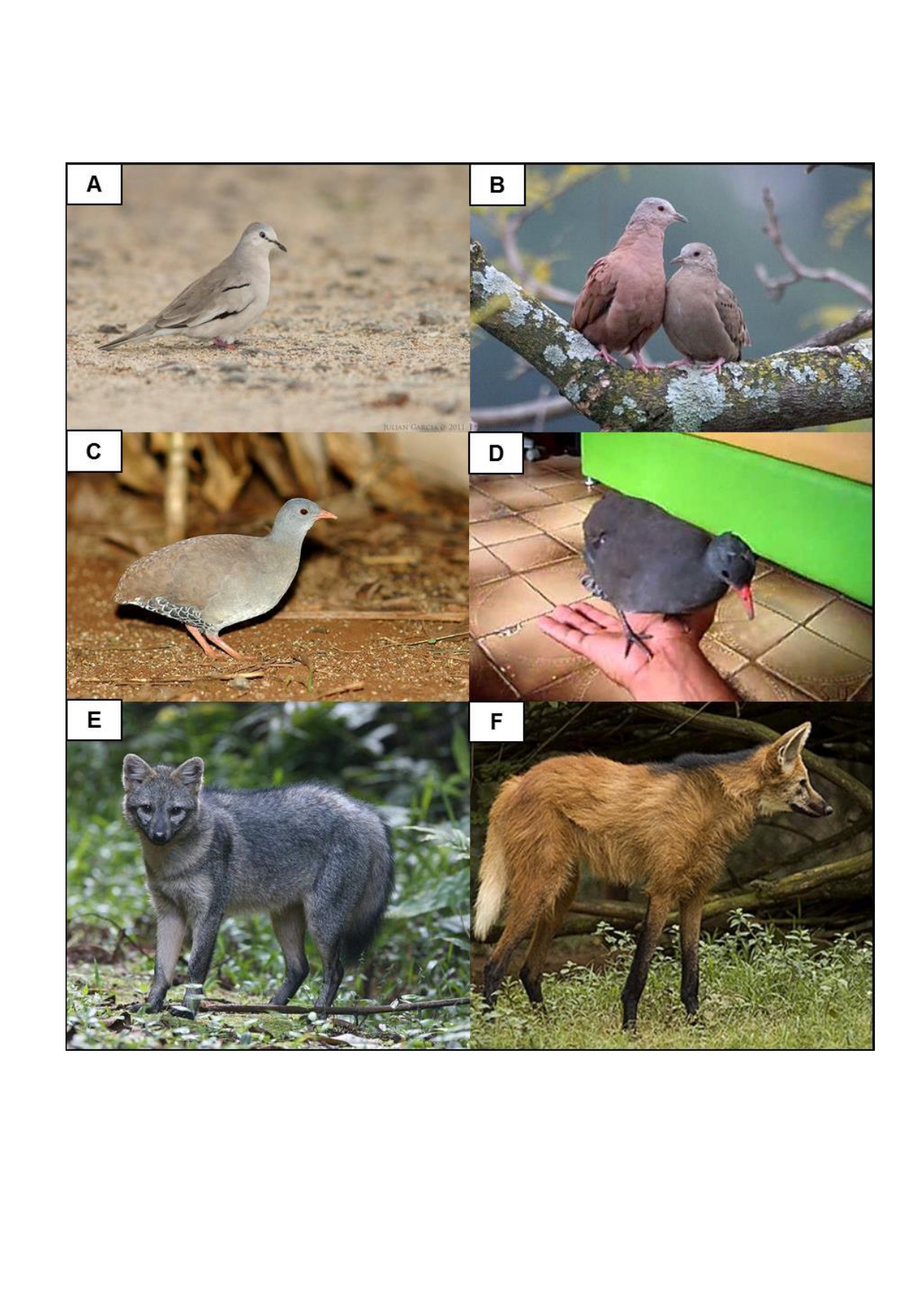

Supplement: Supplementary file 1 — Additional file 1. A checklist containing images of the possible species cited by the interviewees [file 13002_2020_367_MOESM1_ESM.jpg]

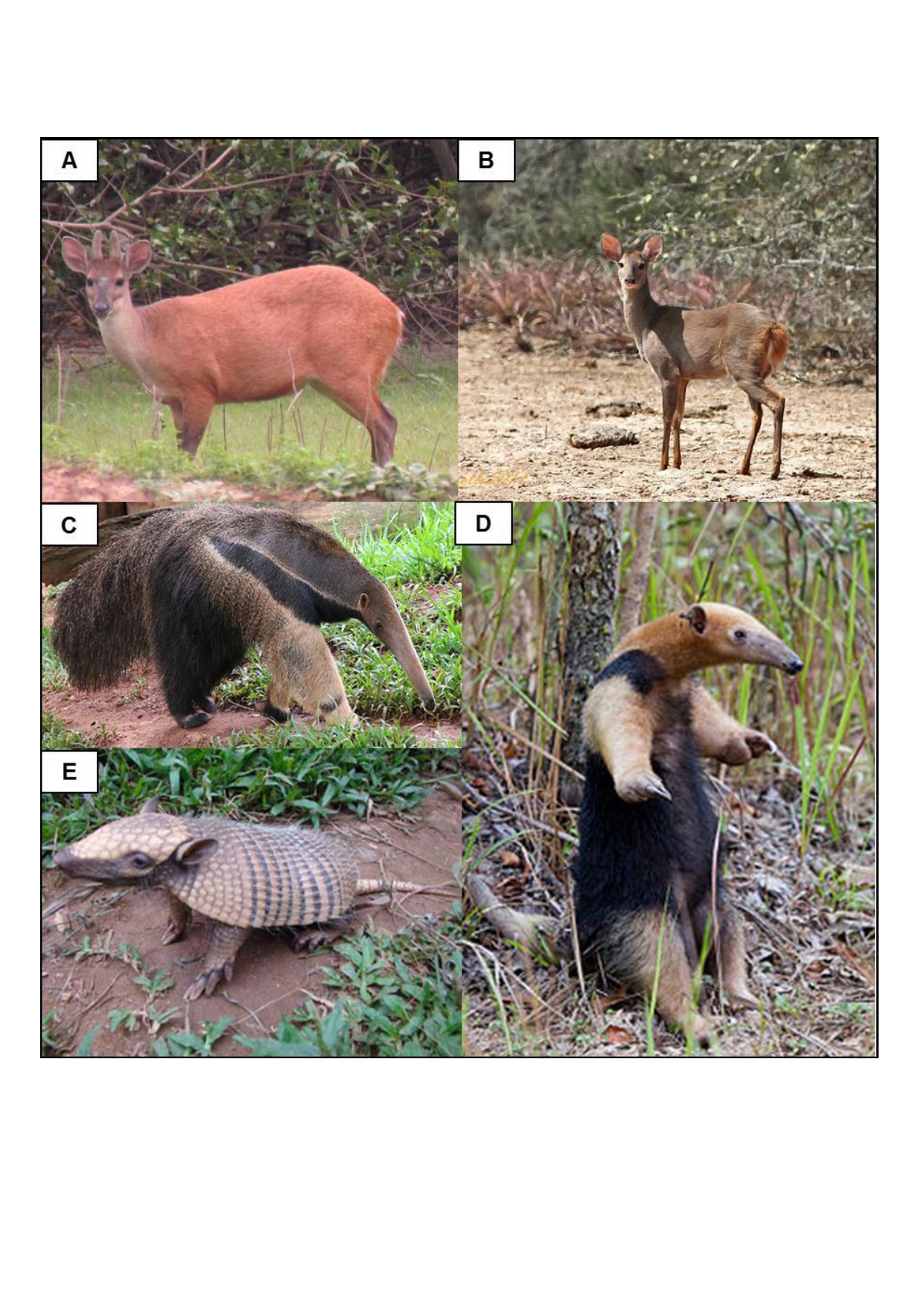

Supplement: Supplementary file 2 — Additional file 2. List of potentially useful game fauna indicated by Fulni-ô people in NE Brazil and conservation status. [file 13002_2020_367_MOESM2_ESM.jpg]

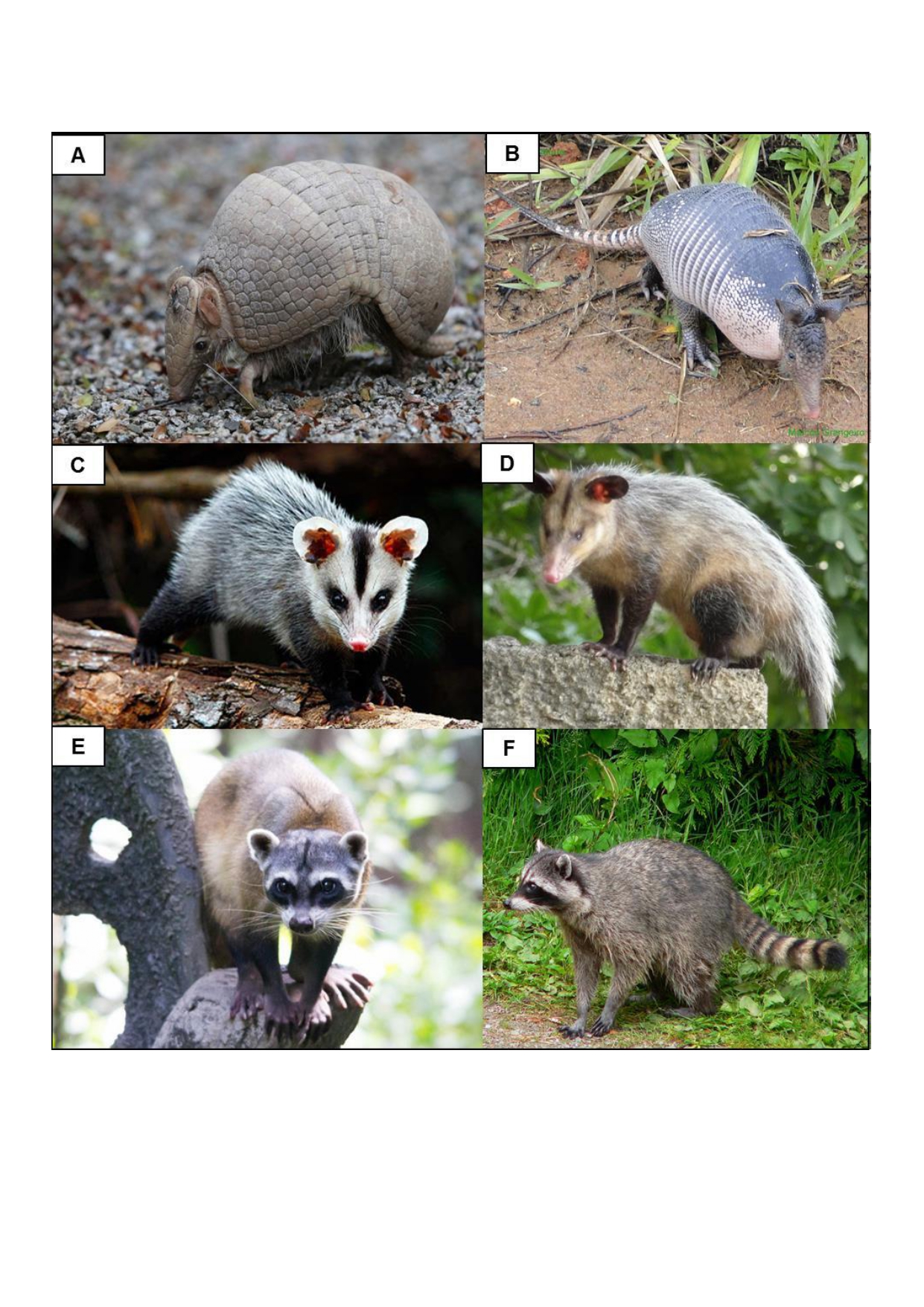

Supplement: Supplementary file 3 — Additional file 3. [file 13002_2020_367_MOESM3_ESM.jpg]

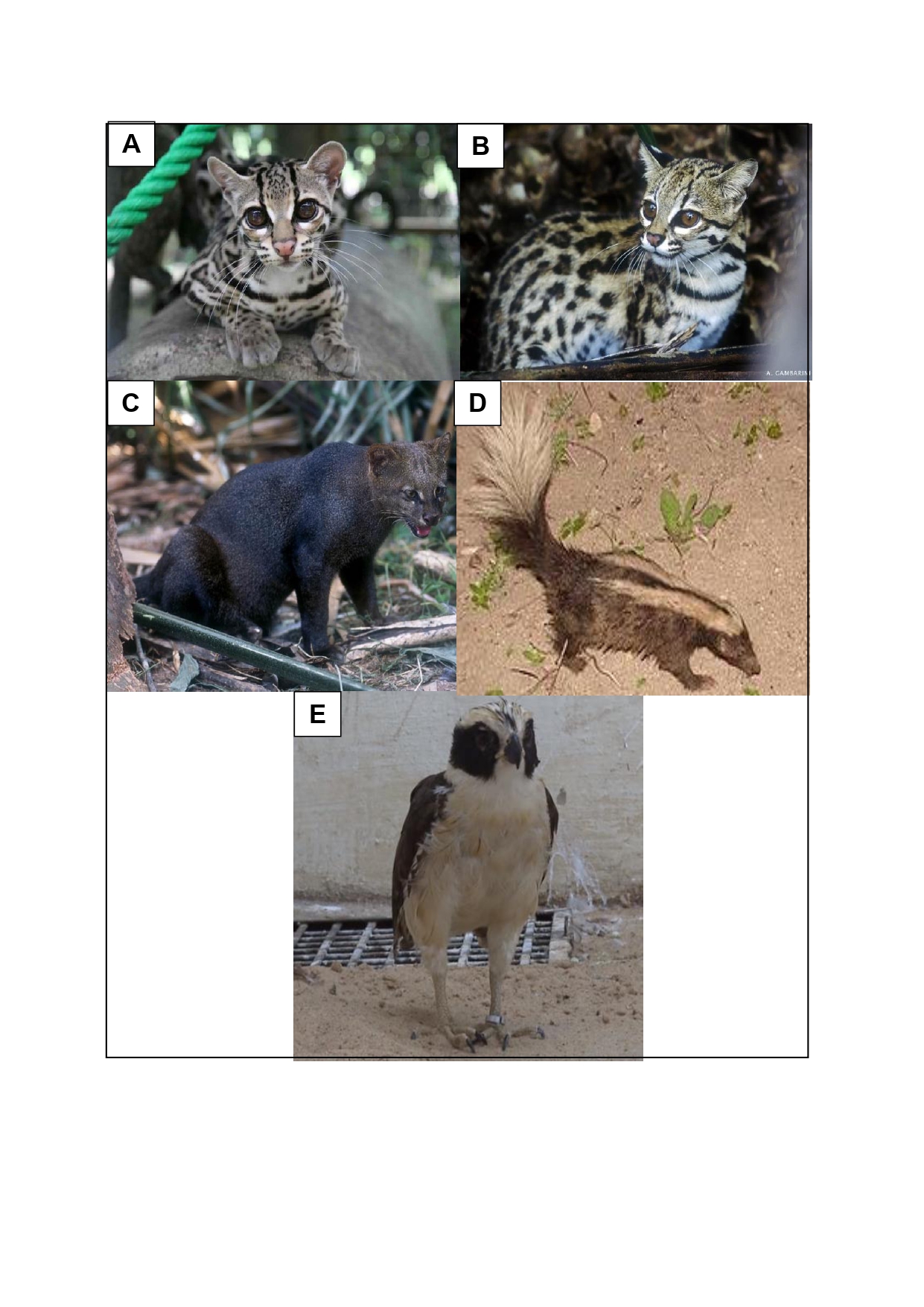

Supplement: Supplementary file 4 — Additional file 4. [file 13002_2020_367_MOESM4_ESM.jpg]

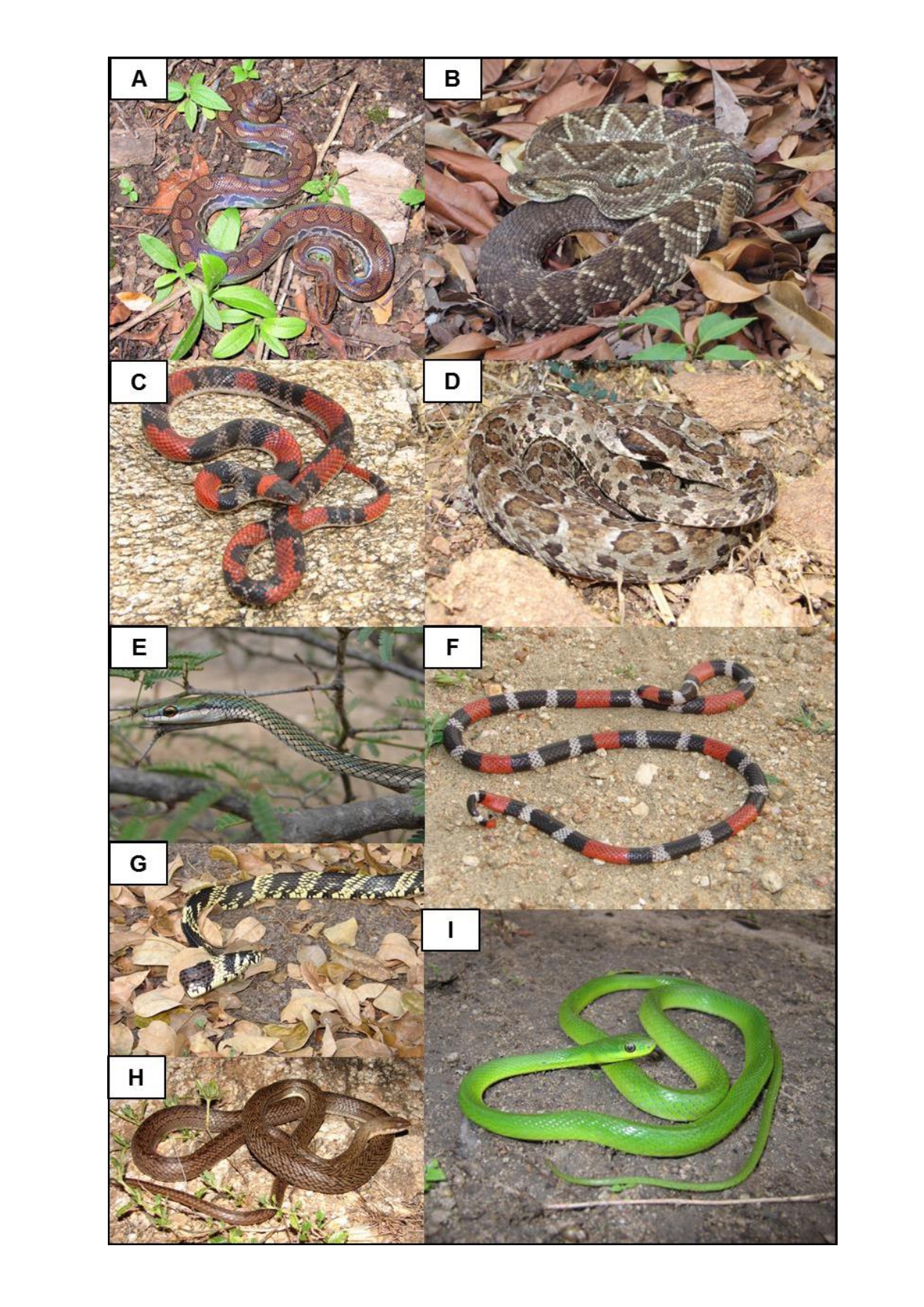

Supplement: Supplementary file 5 — Additional file 5. [file 13002_2020_367_MOESM5_ESM.jpg]
